# High-efficiency crystalline white organic light-emitting diodes

**DOI:** 10.1038/s41377-024-01428-y

**Published:** 2024-04-08

**Authors:** Yijun Liu, Feng Zhu, Yue Wang, Donghang Yan

**Affiliations:** 1 State Key Laboratory of Polymer Physics and Chemistry, Changchun Institute of Applied Chemistry, Chinese Academy of Sciences, Changchun, 130022 China; 2 School of Applied Chemistry and Engineering, University of Science and Technology of China, Hefei, 230026 China; 3 State Key Laboratory of Supramolecular Structure and Materials, Jilin University, Changchun, 130012 China

**Keywords:** Organic LEDs, Optical materials and structures

## Abstract

Crystalline white organic light-emitting diodes (C-WOLEDs) are promising candidates for lighting and display applications. It is urgently necessary, however, to develop energy-saving and high-efficiency C-WOLEDs that have stable and powerful emission to meet commercial demands. Here, we report a crystalline host matrix (CHM) with embedded nanoaggregates (NA) structure for developing high-performance C-WOLEDs by employing a thermally activated delayed fluorescence (TADF) material and orange phosphorescent dopants (Phos.-D). The CHM-TADFNA-D WOLED exhibit a remarkable EQE of 12.8%, which is the highest performance WOLEDs based on crystalline materials. The device has a quick formation of excitons and a well-designed energy transfer process, and possesses a fast ramping of luminance and current density. Compared to recently reported high-performance WOLEDs based on amorphous material route, the C-WOLED achieves a low series-resistance Joule-heat loss ratio and an enhanced photon output, demonstrating its significant potential in developing the next-generation WOLEDs.

## Abstract (web summary)

We report a crystalline host matrix with engineered nanoaggregate structure for fabricating high-performance crystalline white OLEDs (C-WOLED), showing its significant potential in developing the next-generation OLED technology.

## Graphical abstract

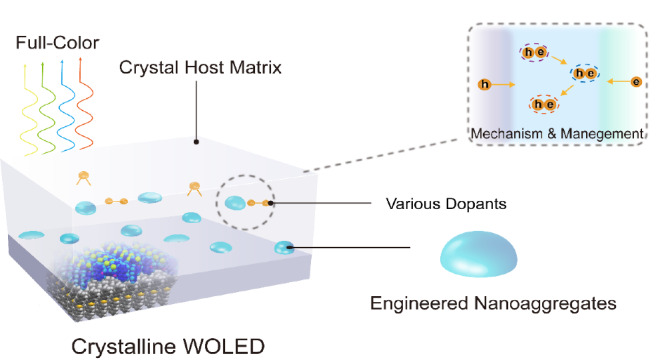

## Introduction

Crystalline organic materials are particularly promising candidates for developing advanced organic light-emitting devices owing to their high carrier mobility and structure stability resulting from the intrinsically long-range ordered molecular arrangement^1–15^. However, since the initial observation of electroluminescence in organic crystals^16^, crystalline organic light-emitting diodes (C-OLEDs) have encountered persistent challenges in achieving precise control over crystalline layers, integrating functional layer, and managing carrier/exciton management and so on. On the contrary, amorphous thin-film OLEDs (A-OLEDs) have achieved successful development into high-performance light-emitting devices. This is attributed to their capability of realizing multilayer device structures and employing delicate doping engineering, which renders facile carrier transport management and efficient exciton utilization^17–19^. Nevertheless, their intrinsic property of disordered molecular arrangement results in low carrier mobility^20^, leading to poor carrier transport and reduce photon output power at low driving voltages.

Recently reported C-OLEDs which adopt polycrystalline thin-film forms are able to combine facile deposition process with the advantages derived from crystalline nature, including high carrier mobility and aligned dipole orientation^5,21–28^. Furthermore, crystalline host matrix (CHM) with embedded nanoaggregates (NA) has emerged as an innovative approach that utilizes both high-mobility properties and high-exciton-utilization-efficiency materials^25^.The CHM-NA architecture is a way to control the luminescence behavior from the perspective of exciton management^29,30^ and realize practicable fabrication engineering for high-performance C-OLEDs.

**Fig. 1:**
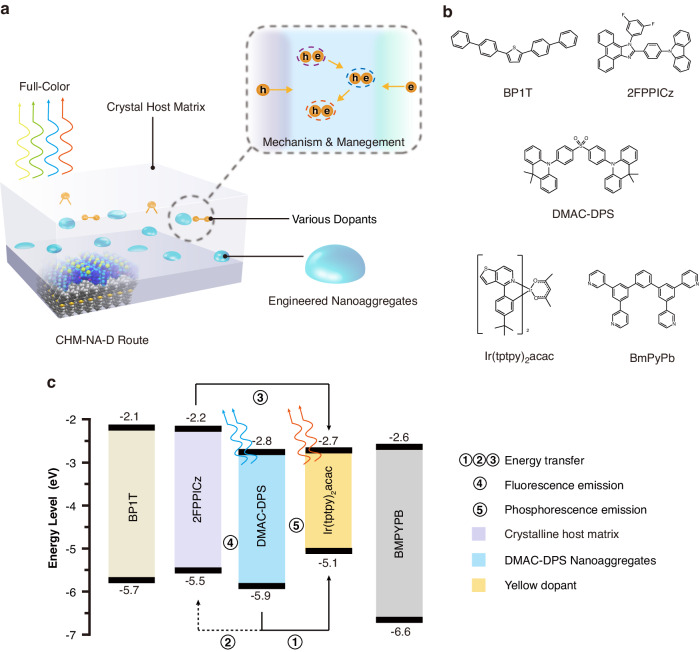
CHM-TADFNA-D structures. **a** Schematic diagram of CHM-TADFNA-D route. **b** Molecular structures of materials. **c** Schematic diagram of energy levels of materials and designed excitons transition process

Figure 1a illustrates the schematic structure of CHM-NA, which can be divided into two modules of CHM and NA. The modules are flexible in their combinations, allowing for various arrangements, including different species of emitting materials and varying numbers of CHM layers. CHM is a crystalline thin film, different from the single crystals, can be prepared by weak-epitaxy-growth (WEG) method during physical vapor deposition^31,32^, In most instances, the electrodes and the single crystal growth process are distinct in crystalline OLEDs. Following the growth of a single crystal on the substrate, it is combined with the electrode using various methods such as wet-transfer^33^, slice epoxy resin method^34^, laminating method^35^, and template stripping^4^. Single crystals excel in high carrier mobility due to their highly ordered alignment of molecules. They are especially suitable for fabrication of Organic Light-Emitting Transistors (OLETs) that offer a great capability of high current densities. Crystalline thin-film OLEDs, produced through the WEG method, have employed vacuum evaporation and are more appropriate for OLEDs with vertical transport characteristics.

While not all organic materials may be compatible with the WEG method for preparing crystalline thin films, high-efficiency guests can serve as NAs on CHM. They can be later formed on the smooth surface of CHM layer during their deposition. CHM with high carrier mobility serves as the foundational host and ensures high carrier transport capability and low driving voltages^23,24,26,27,36^. Meanwhile, amorphous NAs can provide multiple channels for the efficient utilization of excitons, resulting in significantly enhanced photon output. Compared to the original doping route^37,38^, CHM-NA maintains a substantial amount of dopants without compromising the continuity of the crystalline thin film^25^. Furthermore, the fabrication of CHM-NA is simplified in comparison with common doping technologies, since the NA layer does not need to be co-doped with the host material. This method allows for the fabrication of full-color devices by controlling morphologies of emitting layers and managing excitons process. Although CHM-NA strategy has been demonstrated to be capable of realizing high-performance blue-emission C-OLEDs^25^, there is a need for the development of corresponding material systems, excitation management mechanisms, and engineering optimization strategies to create White OLEDs (WOLEDs). In contrast to conventional fluorescent emitters, thermally activated delayed fluorescence (TADF) materials exhibit the ability to convert triplet excitons to singlet excitons through the process of reverse intersystem crossing (RISC) owing to the small ∆E_ST_ (energy gap between the S_1_ and T_1_ states). This characteristic theoretically allows for 100% exciton utilization^39–41^. In the context of WOLEDs, blue TADF emitters have the potential to replace traditional fluorescent materials^42–45^, resulting in hybrid WOLEDs with efficiencies comparable to those of all-phosphorescent WOLEDs. This substitution could offer benefits in terms of both high efficiency and stability^46–49^. The addition of TADF materials in C-OLEDs presents a promising avenue for more efficient routes in fabricating high-performance WOLEDs.

In this work, a novel CHM-NA structure for a WOLED is proposed, which combines a fluorescent CHM with blue TADF nanoaggregates (TADFNA) and orange phosphorescent dopants (Phos.-D). The high carrier mobility generated from CHM brings advantages of low driving voltages, while the high-efficiency emitters of nanoaggregates and dopants achieve remarkable efficiencies through the full utilization of excitons. A hybrid C-WOLED with a low turn-on voltage of 2.7 V (@ 1 cd m^−2^), a maximum power efficiency of 43.3 lm W^−1^, a maximum current efficiency of 38.6 cd A^−1^ and a maximum external quantum efficiency (EQE) of 12.8% have been fabricated. These results represent the highest performance among WOLEDs based on organic crystalline materials. The low operation voltage as well as the high photon output efficiency of the WOLED can be attributed to the careful design, well-engineered crystalline structures and efficient utilization of excitons.

## Results

### Preparation of CHM and nanoaggregates

Figure 1 depicts the fundamental concept of the white-emission C-OLED proposed in this work. Figure 1b shows the molecular configurations and energy levels of materials used in the construction of CHM-TADFNA-D WOLEDs. The device was fabricated by vacuum deposition under a pressure of 10^−4^ Pa. Serving as both a hole injection layer (HIL) and a flat surface for the following crystal growth, the indium tin oxide (ITO) anode was coated with a 40-nm thick poly(3,4-ethylenedioxythiophene) polystyrene sulfonate (PEDOT:PSS) layer.

The main crystalline thin films were grown by WEG method, allowing for precise control of films’ thickness. A rod-like 7-nm thick 2,5-di([1,1’-biphenyl]-4-yl)thiophene (BP1T) crystalline thin film was grown on PEDOT:PSS to induce the oriented growth of the crystalline hole transport layer and emission layers^24,50^. For the emission layer, a fluorescent deep-blue material with high carrier mobility, 2-(4-(9H-carbazol-9-yl)phenyl)-1-(3,5-difluorophenyl)-1H-phenanthro[9,10-d]imidazole (2FPPICz)^51^, was grown to serve as both hole transport layer (7-nm) and CHM (5-nm). Subsequently, the blue TADF material, bis[4-(9,9-dimethyl-9,10-dihydroacridine)phenyl]sulfone (DMAC-DPS)^52^, was sequentially deposited on 2FPPICz layer for 1-nm, forming uniform embedded nanoaggregates. The CHM-NA structure was repeated four times to fabricate a multilayer structure. The orange phosphorescent material, iridium(III)bis(4-(4-t-butylphenyl)thieno[3,2-c]pyridinato-N,C2′)acetylacetonate (Ir(tptpy)_2_acac) was doped (5 wt%) into the third five-nanometer region of 2FPPICz CHM by co-evaporate with it. Afterwards, amorphous electron transport layer, electron injection layer, and cathode were fabricated on the crystalline emission layer, consisting of 10-nm 1,3-bis(3,5-dipyrid-3-yl-phenyl)benzene (BmPyPb), 40-nm BmPyPb, 1-nm LiF and 150-nm Al.

The basic design of exciton transition processes in CHM-TADFNA-D WOLED is presented in Fig. 1c. According to our previous results and expectations^25^, excitons will firstly form in DMAC-DPS nanoaggregates. Other than the doped device, the exposed top parts of nanoaggregates on the film surface can facilitate the direct injection of electrons from the electron transport layer into nanoaggregates^25^. Considering that the undoped DMAC-DPS OLED achieves efficient blue emission similar to doped ones^53^, the blue nanoaggregates will exhibit a high efficiency rather than aggregation-caused quenching. Although the low triplet state of the 2FPPICz CHM may result in a leakage of triplet excitons, they can be reused during an energy transfer process by an orange phosphorescent material with a lower triplet state. This characteristic allows for the fabrication of a high-efficiency C-WOLED.

**Fig. 2:**
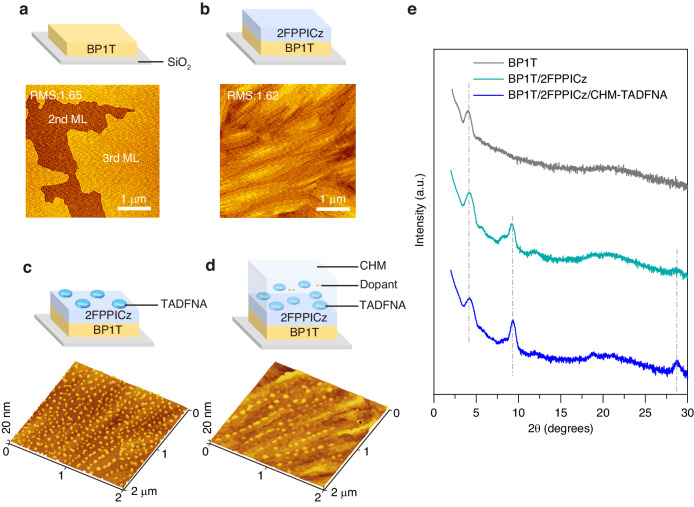
Schematic illustrations and AFM images of crystalline thin film and nanoaggregates. **a** BP1T crystalline thin film, **b** 2FPPICz crystalline epitaxy thin film. Schematic illustrations and 3D AFM images of (**c**) DMAC-DPS nanoaggregates grown on 2FPPICz crystalline epitaxy thin film, **d** 2FPPICz crystalline host including DMAC-DPS nanoaggregates with Ir(tptpy)_2_acac 1 wt%. **e** Out-of-plane XRD patterns of BP1T crystalline thin film, 2FPPICz crystalline host matrix and 2FPPICz crystalline host matrix including DMAC-DPS nanoaggregates with Ir(tptpy)_2_acac 1 wt%

To achieve high carrier mobility, it is crucial to fabricate devices with a high degree of order and orientation. As mentioned above, CHM has been grown by WEG, enabling precise control over the thickness of each organic layer and the preparation of large-area, continuous, single-crystal-like polycrystalline thin films^36,54^. A schematic diagram and morphologies of each CHM-TADFNA portion are shown in Fig. 2a–d. CHM crystallized as stripe-like crystal bars, then amorphous nanoaggregates were formed on top of them. The scanning electron microscope (SEM) image (Fig. S2) also show oblate ellipsoid like nanoaggregates on flat CHM. Additionally, the morphologies morphological analysis reveals that DMAC-DPS nanoaggregates can grow on crystalline 2FPPICz substrates without compromising the quality of epitaxial film growth. This is evident as 2FPPICz can continuously grow along in areas where nanoaggregates are absent. Figure 2e shows the out-of-plane X-ray diffraction (XRD) patterns of the three kinds of WEG crystalline thin films. The gray and green solid lines, corresponding to BP1T crystalline thin film and BP1T/2FPPICz crystalline film, respectively, accord with the previously reported BP1T and 2FPPICz results^24^. On the other hand, the blue solid line corresponding to the thin film with nanoaggregates does not show any additional peaks, while the peaks of BP1T and 2FPPICz still remain unchanged.

### Performance of CHM-TADFNA-D WOLED

It has been demonstrated that 2FPPICz WEG thin film possesses hole and electron mobilities of ~0.10 and 0.015 cm^2^ v^−1^ by time-of-flight method, respectively, owing to its unique crystalline structures^24^. As mentioned before, CHM-TADFNA thin film can remain a high-crystallinity morphology, which is the precondition for the fabrication of high-efficiency C-OLEDs. As high-performance blue emission is fatal precursor to WOLEDs, a reference blue CHM-TADFNA Device (Device B), ITO/PEDOT:PSS (40 nm)/BP1T (7 nm)/2FPPICz (7 nm)/EML 1 (24 nm)/BmPyPb (40 nm)/LiF/Al was first fabricated and the device structures are shown in Fig. S1.

To determine the advantages brought by CHM-NA structure, Fig. 2a–d and Table 1 show the main characteristic of Device B and the originally reported amorphous DMAC-DPS device^53^. The CHM-TADFNA device exhibits a low turn-on voltage of about 2.7 V, an emission peak at 471 nm with a full width at half maximum of ~63 nm, a maximum EQE of 7.22% and a Commission International de L’Eclairage (CIE) value of (0.16, 0.20) at 1000 cd m^−2^. The detailed optimization of Device B is reported separately^55^. In comparison with reported amorphous device, CHM-TADFNA device exhibits a lower turn-on voltage, and both the luminance and current density experience a rapid ramping at low driving voltages. The climb in brightness from 1 cd m^−2^ to 1000 cd m^−2^ (∆*V*) requires only 1.6 V, indicating a much faster rise. The low operation voltage is a desirable characteristic for practical applications.

**Table 1:** Summary of EL performances of the CHM-TADFNA OLED and amorphous devices

Device	*V*_on_^a^ [V]	*V*_1000_^b^ [V]	∆*V*^c^ [V]	*λ* [nm]	FWHM [nm]	EQE_max_^d^ [%]	CIE(*x*, *y*)^e^
CHM-TADFNA	2.7	4.3	1.6	471	63	7.22	0.15, 0.20
Amorphous 1	2.9	4.9	2.0	479	77	11.8	0.16, 0.29
Amorphous 2	3.0	5.0	2.0	481	77	14.4	0.17, 0.30

^a^*V*_on_ is the turn-on voltage which is measured at 1 cd m^−2^

^b^*V*_1000_ is operation voltage at the luminance of 1000 cd m^−2^

^c^∆*V* is the difference between *V*_on_ and *V*_1000_

^d^EQE_max_ correspond to maximum external quantum efficiency values

^e^Commission Internationale de L’Eclairage (CIE) coordinates at luminance of 1000 cd m^−2^

**Fig. 3:**
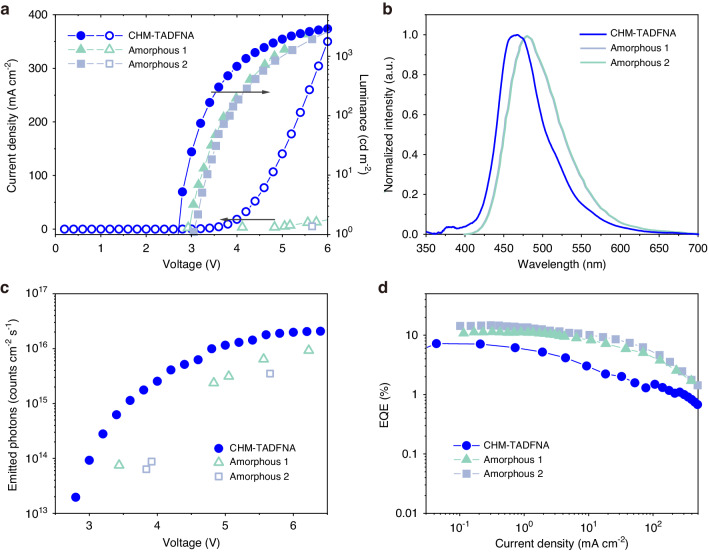
CHM-TADFNA OLEDs. **a** Voltage-dependent current density and luminance. **b** EL spectrum at 1000 cd m^−2^. **c** Semi-log emitted photons (*N*), between the CHM-TADFNA OLED and reported OLEDs based on DMAC-DPS^50^. All reference data for comparison are extracted from the corresponding literature. **d** EQE-current density characteristics

Due to human-eye visual function will influence the evaluation of OLEDs, the quantity of emitted photons per unit time per unit area (*N*) is a better evaluation standard rather than luminance. Therefore, the impact of the human visual function can be excluded. *N* is acquired from the current density (*J*) and the EQE, calculating from *N* = EQE*J/e, where *e* represents the elementary charge. *N* will increase with the growth of EQE and *J*. In other words, the higher EQE and conductance are, the larger photon output there will be. Figure 3c illustrates that CHM-TADFNA device exhibits much enhanced photoemission than those amorphous devices, due to the high carrier mobility of crystalline thin films. This high mobility enables excitons to form much more rapidly.

In addition, the EL spectrum of CHM-TADFNA appear a blue shift and narrowing, indicating the potential of CHM-TADFNA structure in fabrication of deep-blue devices. The blue CHM-TADFNA device have an EQE value of 7.22%. Although it exceeds the limitation of 5% compared to fluorescent materials and indicates TADF process to some extent, the EQE is still lower than those of reported doped^52^ and undoped^53^ devices based on DMAC-DPS. It is speculated that the lower triplet state of the CHM (*T*_1_^C^ = 2.49 eV^51^) may have contributed to the lower external quantum efficiency (EQE) of the blue CHM-TADFNA device. As shown in Fig. S3a, b, to understand the energy transport process, we determined the distribution of excitons in CHM-NA device (Device C①) and pure crystalline device (Device C②). The distribution schemes of excitons at different voltages are shown in Fig. S3h, k. At 3.4 V, the EL spectrum of Device C② cannot be detected, while Device C① shows strong luminescence. Due to the fact that the exciton distributions have not changed significantly, the change in spectrum results from the unnecessary transfer of energy from TADFNA back to CHM. The EL spectrum in Fig. S4 also proved the result. The EL spectrum remains stable at 1000 cd m^−2^, but at high voltages, strong 2FPPICz emissions at 390, 408 and 402 nm can be observed.

**Fig. 4:**
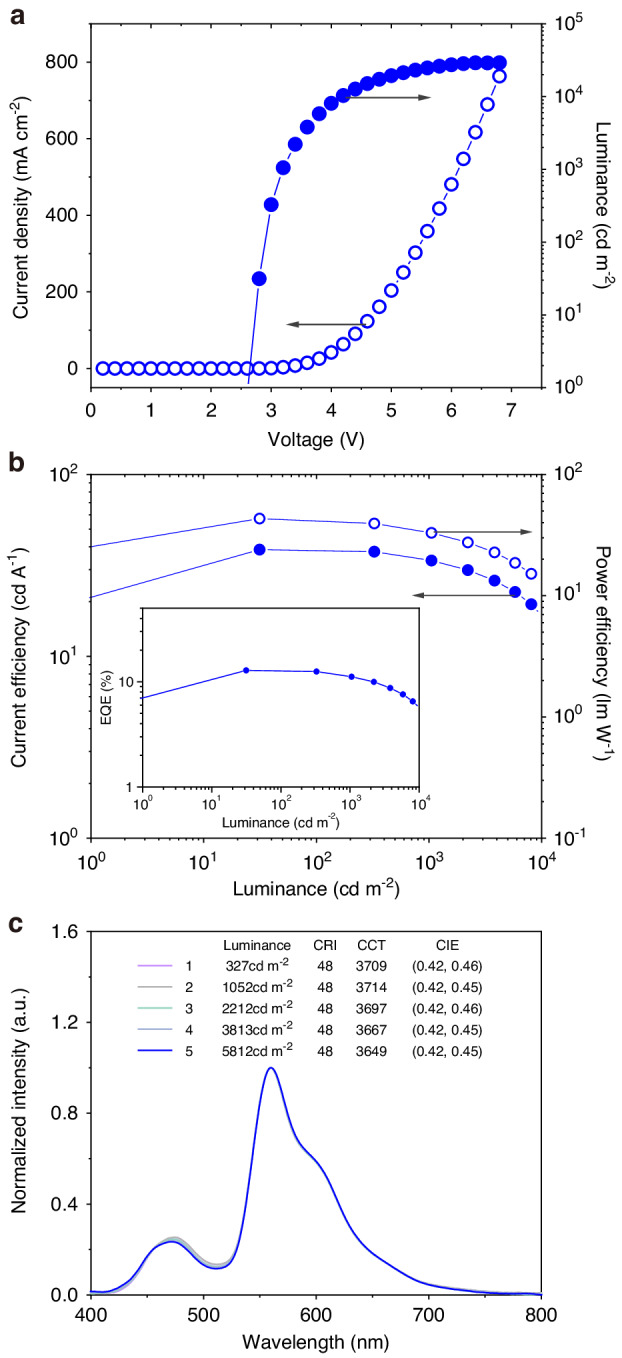
CHM-TADFNA-D WOLEDs. **a** Voltage-dependent current density and luminance. **b** Luminance dependent CE and PE characteristics, the inset is EQE-luminance characteristics. **c** EL spectrum at varied luminance

Conceivably, if there is a high-efficiency phosphorescent material that can harvest the energy leaked from TADFNA instead of allowing its transfer to CHM, the EQE of the device will increase substantially. This encourages us to further design CHM-TADFNA-D WOLEDs based on the CHM-TADFNA device. Figure 4a–d shows the EL performance of the optimized CHM-TADFNA-D WOLED, which is defined as Device W. In consistence with the blue CHM-TADFNA device, the optimized Device W shows impressive features. It has a turn-on voltage of about 2.7 V. And the luminance and current density have a rapid growth at low driving voltages, resulting in a luminance of 1000 cd m^−2^ at 3.2 V. The impressive characteristics in voltage lead to low operating voltages and ∆*V* (the difference between *V*_on_ and *V*_1000_) of 0.5 V. Figure 3c exhibits a stable spectral property when the CIE coordinates change slightly from (0.42, 0.46) to (0.42, 0.45) with the luminance goes from 327 cd m^−2^ to 5812 cd m^−2^. It is the first time that WOLED based on crystalline materials can exhibit stable spectral characteristics, which is a rigid requirement in practical applications. The maximum forward-viewing EQE, current efficiency (CE), and power efficiency (PE) are 12.8%, 38.6 cd A^−1^, 43.3 lm W^−1^, respectively, and they remain 11.2%, 33.6 cd m^−2^, 33.0 lm W^−1^ at the practical brightness of 1000 cd m^−2^. These results achieved an evident improvement compared to previously reported WOLEDs based on crystalline materials, and even comparable to those amorphous ones.

### Analysis of device mechanism

**Fig. 5:**
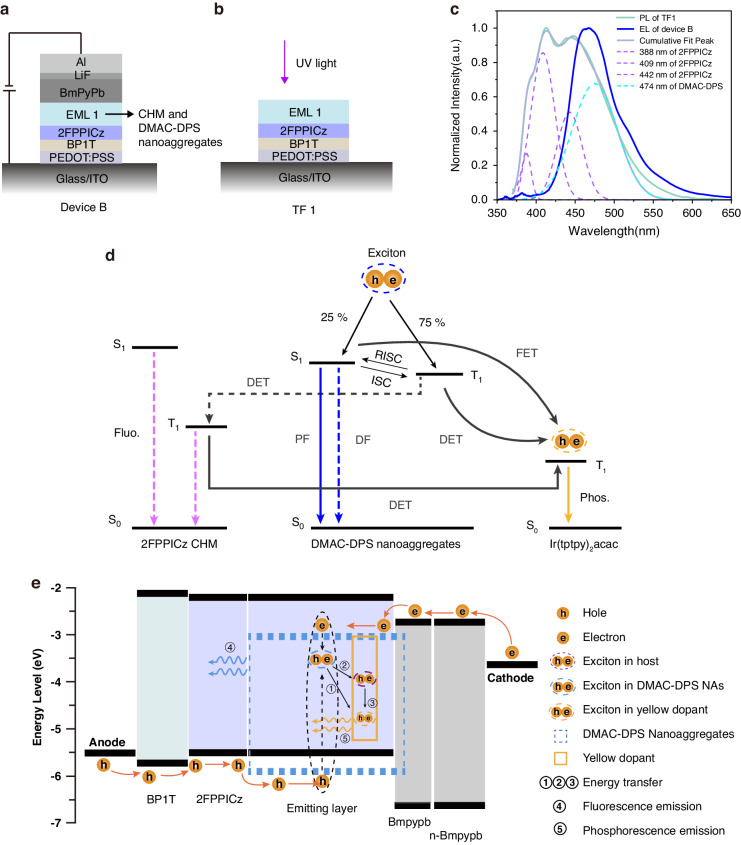
Mechanism of CHM-TADFNA-D WOLED. **a** Structure of device B. **b** Structure of TF 1. UV ultraviolet. **c** Comparison of the EL of Device B and the PL of TF 1, the dash lines represent fitted peaks. **d** Schematic diagram of the energy transport processes of CHM, nanoaggregates and dopants. **e** Schematic illustration of the energy-level diagram and working mechanism of the CHM-TADFNA-D WOLED

To further investigate the mechanism of these CHM-TADFNA devices, we determined the formation and transport process of excitons. To demonstrate the excitons behavior, a thin film structure TF 1 (Fig. 5b), Quartz substrate/BP1T (7 nm)/2FPPICz (7 nm)/EML2 (24 nm) was fabricated. As mentioned before, BP1T and 2FPPICz grew by the method of WEG to form crystalline thin films. EML 2 was composed of four loops of 1 nm thick DMAC-DPS nanoaggregates and 5 nm thick 2FPPICz CHM without a yellow phosphorescent dopant. The photoluminescence (PL) spectrum of TF 1 was shown in Fig. 5c. The green PL spectrum line shows three apparent characteristic peaks at 388 nm, 409 nm and 442 nm of 2FPPICz after peak splitting. In comparison, the deep-blue line in Fig. 5c shows EL spectrum of Device B at about 1000 cd m^−2^, which only reveal emission of DMAC-DPS. Figure S4 exhibits spectrum at other voltages, revealing apparent peaks of 2FPPICz.

These results indicate that in Device B, excitons form directly on DMAC-DPS nanoaggregates rather than on 2FPPICz CHM, as indicated by the absence of the EL spectrum of 2FPPICz. Additionally, the turn-on voltage of the Device B is about 2.7 V while the pure crystalline 2FPPICz device usually have a turn-on voltage of 3.0 V^24^. Meanwhile, a Device D, ITO/PEDOT:PSS (40 nm)/BP1T (7 nm)/2FPPICz (7 nm)/EML3 (20 nm)/BmPyPb (40 nm)/LiF/Al, was fabricated. 2FPPICz was still crystalline thin films and EML 3 was consisted of 20 nm thick amorphous DMAC-DPS. The performance of Device D is illustrated in Fig. S4a–d, whose turn-on voltage is 2.9 V, instead. Obviously, the current density of Device B is higher than those of Device D and reported amorphous devices (Fig. 3a) at same driving voltages^53^. Moreover, the turn-on voltage of Device B (2.7 V) is lower than those of Device D (2.9 V) and reported amorphous devices (2.9 V to 4.3 V)^53^. It can be inferred that Device B exhibits higher conductance and faster turn-on characteristic, attributing to CHM-NA-D route.

Based on the obtained results, it can be concluded that the coexistence of CHM and nanoaggregates contributes to both high conductance and low turn-on voltage. The conductance is determined by charge concentration and carrier mobility. While CHM has been demonstrated to have a high carrier mobility in previous work^24^, it is crucial to assess whether the charge concentration is affected by the presence of the CHM-TADFNA route. To demonstrate the possible increase in charge concentration, we verify the heterojunction effect, which facilitates charge transfer between DMAC-DPS nanoaggregates and crystalline 2FPPICz film.

As Fig. S6a shows, four in-plane transport devices were fabricated. Figure S6b shows the measured current under electrical fields in these four devices. It is clear that only the Device E④ with 2FPPICz on top exhibits an evident ohmic-like behavior. The results confirm that there is a heterojunction between DMAC-DPS and 2FPPICz film. The conduction of Device E④ is two orders of magnitude higher than that of Device E②, indicating a significantly enhanced heterojunction effect. Given the superior hole transport capability of 2FPPICz, it can be assumed that when DMAC-DPS contacts 2FPPICz, charge transfer occurs, resulting in hole accumulation and an increase in current density. Meanwhile, electrons accumulate in DMAC-DPS nanoaggregates and eventually lead to a band bending of 2FPPICz/DMAC-DPS heterojunction, causing an increase in carrier concentration. A similar model has been proved in our previous research^25^.

Despite the fact that the excitons in DMAC-DPS nanoaggregates can utilize triplet excitons via RISC process, the CHM, with a lower triplet energy level, will absorb a portion of them, leading to a wastage and an unstable spectrum (Fig. S4). Nonetheless, Device W shows a stable EL spectrum, suggesting that energy has been transferred between the phosphorescent material and excitons of DMAC-DPS rather than being wasted. Additionally, the doping position of the CHM-TADFNA-D WOLED also plays an important role in its stability (Fig. S7). To further investigate the mechanisms, we determined the distribution of excitons in Device B (Fig. S8). According to Fig. S8f, the excitons concentrate in the range of the third five-nanometer region for Device B. Based on the exciton recombination zone, it can be concluded that the phosphorescent material doped in the third five-nanometer region can assimilate the majority of redundant triplet excitons from DMAC-DPS, which results in a stable spectrum. Furthermore, Device W① and Device W② exhibit increased blue light emission at high driving voltages while the Device W and Device W③ emits less blue light under similar conditions, which suggests different mechanisms. Energy transfer and charge carrier trapping are two basic mechanisms in OLEDs^56,57^. It reveals that for Device W① and Device W②, the orange emission is majorly originated from charge trapping, due to the hole trapping of phosphorescent material^58^. Although Device W③ shares similar characteristics with Device W, it fails to efficiently capture most other excitons in third five-nanometer region, resulting in a changeable spectrum. However, for Device W, a significant portion of the orange light is emitted through energy transfer, by which the excess triplet excitons in CHM can be reused. The relatively stable spectrum can be attributed to the well-distributed doped phosphor.

To demonstrate more detailed EL processes in the CHM-TADFNA-D WOLED, a series of transient EL decay devices was fabricated. Except for EMLs, these devices share the same structures as the previously mentioned CHM-TADFNA structures. Device F1 mirrors the structure of Device B mentioned before. Device F2 and Device F3 closely resemble Device W, differing only in the doping concentration. Specifically, Device F2 is doped with 1% Ir(tptpy)_2_acac, while Device F3 has 10% doping into the third five-nanometer region.

Figure S9a, b shows their EL decay curves measured at 471 nm and 555 nm, which correspond to the emission peaks of DMAC-DPS nanoaggregates and Ir(tptpy)_2_acac. Obviously, when the electrical pulse excitation was turned off, Device F2 exhibited a delayed component compared to Device F1 at the wavelength of 471 nm. It reveals that the CHM-TADFNA structure will not influence the TADF properties of materials and explain why the EQE of Device B can exceed 5%. Additionally, Fig. S9a shows the delayed curves of Device F2 and Device F3 measured at 471 nm, revealing a decreasing tendency. The result indicates an energy transfer process from TADF-NA to Ir(tptpy)_2_acac dopants.

As further evidence of energy transfer process, Fig. S8b shows delayed curves measured at 555 nm. Due to the enhanced energy transfer transition to Ir(tptpy)_2_acac dopants, the delayed lifetime simultaneously increases with higher doping concentrations. These results are consistent with our prior expectation, Ir(tptpy)_2_acac will harvest triplet excitons in CHM through an efficient energy transfer process, preventing unnecessary exciton quenching in 2FPPICz CHM. Figure 5d illustrates the energy transfer mechanism in CHM-TADFNA-D WOLED.

Moreover, in accordance with previous discussions regarding carrier transportation, Fig. 5e illustrates the energy-level and mechanism of CHM-TADFNA-D WOLED. The hole and electron carriers pass via anode and cathode, then encounter at DMAC-DPS nanoaggregates. Due to the high carrier mobility of CHM, excitons can form rapidly. The DMAC-DPS will harvest the generated triplet excitons by a RISC process due to a small singlet-triplet energy gap (ΔE_ST_) according to the previous report^53^. Some excitons directly emit blue light, while others will transport to the doped phosphor by the energy transfer process. Through this approach, a highly efficient hybrid CHM-TADFNA-D WOLED is realized.

### Photon output characteristic

**Fig. 6:**
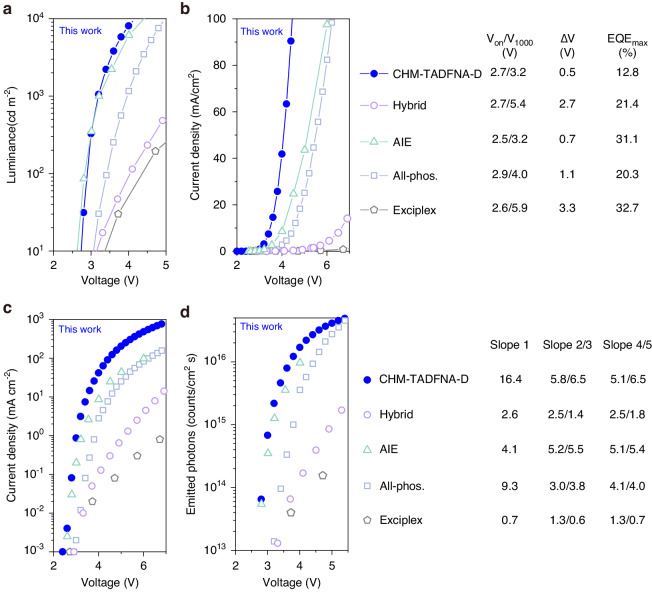
Comparisons of CHM-TADFNA-D WOLED with amorphous WOLEDs. **a** A comparison of voltage (*V*)-luminance and (**b**) voltage (*V*)-dependent current density (*J*). **c** Comparison of voltage-dependent semi-log density. **d** Comparison of voltage(*V*)-dependent semi-log emitted photons (*N*), between the CHM-TADFNA-D WOLED and reported WOLEDs, including hybrid WOLED, AIE WOLED, all-phosphorescent WOLED and exciplex WOLED. All reference data for comparison are extracted from the corresponding literature

Further, as we discussed before, the high carrier mobility of crystalline structures will result in an increase in light output, and the formation of excitons will be faster than in amorphous devices, leading to high-yielding excitons. Therefore, CHM-TADFNA-D WOLED is able to enhance photo emission at the same driving voltages, which is in urgent demand of practical application. Figure 6a–d and Table 2 present a comparison between the CHM-TADFNA-D WOLED and reported state-of-the-art amorphous WOLEDs with high EQE, including hybrid WOLED (fluorescence blue materials and phosphorescent yellow materials), AIE WOLED, all-phosphorescent (Phos.) WOLED and exciplex WOLED^59–62^. A comparison with other WOLEDs based on crystalline materials^7,63^ is also shown in Fig. S10a–d. The CHM-TADFNA-D WOLED exhibits advantages in luminance, current density, turn-on/driving voltages, and photon emission properties.

**Table 2:** Comparison of CHM-TADFNA WOLED and with amorphous WOLED

Device	*V*_on_/*V*_1000_/∆*V* [V]	PE_max_/PE_1000_^a^ [lm W^−1^]	Input power [mW cm^−2^]	Joule heat [mW cm^−2^]	Ratio [%]	CIE^b^ (*x*, *y*)	Ref.
This work	2.7/3.2/0.5	43.3/38.6	10.02	0.60	5.97	0.42, 0.45	This work
Hybrid	2.7/5.4/2.7	69.7/50.6	7.99	0.85	10.60	0.44, 0.45	^57^
AIE	2.5/3.2/0.7	130.7/78.5	2.57	0.15	6.00	0.37, 0.50	^58^
All-phos.	2.9/4.0/1.1	39.0/42.1	11.21	0.84	7.50	0.47, 0.43	^56^
Exciplex	2.6/5.9/3.2	108.2/63.6	5.38	0.91	16.94	–	^59^

All reference data for comparison are extracted from the corresponding literature

^a^PE_max_ is the maximum power efficiency and PE_1000_ is power efficiency at the luminance of 1000 cd m^−2^

^b^Commission Internationale de L’Eclairage (CIE) coordinates at luminance of 1000 cd m^−2^

As shown in Fig. 6a, the luminance of CHM-TADFNA-D WOLED surpasses that of representative amorphous ones under different driving voltages. Obviously, the slope of CHM-TADFNA-D WOLED is the biggest, revealing the lowest driving voltage and fastest increase velocity. The luminance switching from 1 cd m^−2^ to 1000 cd m^−2^ only needs 0.5 V, which is the smallest value among all the reported WOLED studies based on amorphous materials in labs as far as we known. This characteristic is crucial for practical WOLED applications. Similarly, Fig. 6b illustrates that the CHM-TADFNA-D WOLED exhibits the fastest ramping in current density, indicating that it has the highest conductance due to its high mobility of crystalline portions.

To further study this phenomenon, we define the instantaneous slope of *J-V* curves at their corresponding luminance of about 1000 cd m^−2^ as Slope 1, i.e., areal differential conductance. Figure 6 summarizes detailed electric parameters, including input power, Joule-heat loss ratio of series resistance, and so on. The series-resistance joule-heat loss ratio, instead of joule heat number, can represent the inevitable energy loss of operating OLEDs^51^. The CHM-TADFNA-D WOLED possesses the highest areal conductance (16.4 mS cm^−2^) and the lowest ratio of series resistance Joule heating to input power (5.97%). The decreased ineluctably energy loss of operating WOLEDs provides more evidence that the CHM-TADFNA-D route can utilize energy efficiently. In Fig. 6c, the semi-log of current density versus voltage curves are showed to research the rate of conductance change at low operation voltages of WOLEDs. The instantaneous slope of the log(*J*)-*V* curves at 1 cd m^−2^ and the average slope from 1 cd m^−2^ to 1000 cd m^−2^ are expressed by Slope 2 and Slope 3, respectively. From Fig. 6, it is clear that the CHM-TADFNA-D WOLED possesses higher Slope 2 and Slope 3, suggesting that high-mobility CHM structure contributes enormously to improving the conductance of WOLEDs and the fast turning-on under low driving voltages. Regarding the emitted photons discussed earlier, Fig. 6d shows that the CHM-TADFNA-D WOLED possesses a higher *N*. Furthermore, Slope 4 and Slope 5 are defined as the instantaneous of log(*N*)-*V* curves at 1 cd m^−2^ and the average from 1 cd m^−2^ to 1000 cd m^−2^. The higher values of slope 4/5 illustrate that the CHM-TADFNA-D WOLED is able to obtain quite high photo emission at rather low operating voltages. WOLEDs show strong photoemission due to the high carrier mobility of the CHM structure and well-utilized excitons.

## Discussion

Crystalline host matrix (CHM) with embedded nanoaggregates (NA) offers a unique combination of the high mobility characteristic of crystalline materials and the efficient exciton utilization of emitting materials. By applying the CHM-NA structure, it is possible to control luminescence behavior in a novel manner and different devices can be created by modulating the components within the structure. Here, we developed a novel type of WOLED by utilizing CHM, blue TADF material nanoaggregates and orange phosphorescence dopants. Nanoaggregates will not influence the continuity of 2FPPICz CHM, therefore the unique superior performance of CHM in mobility can be reserved. The CHM-TADFNA-D strategy guarantee high mobility as well as high utilization of excitons, ensuring the fast formation of excitons and a delicately designed energy transfer process. It possesses a fast turn-on at low driving voltages and a fast ramping of luminance with voltages. Furthermore, the device achieves a promising low ratio of series-resistance Joule heating to input power and a strongly enhanced photo output. The CHM-TADFNA-D route provides a practical solution to fabricate high-performance WOLEDs and expand the range of application of CHM-NA structures. The CHM-TADFNA-D exhibits a turn-on voltage of 2.7 V (@ 1 cd m^−2^), a maximum power efficiency of 43.3 lm W^−1^, a maximum current efficiency of 38.6 cd A^−1^ and a maximum external quantum efficiency (EQE) of 12.8%, which are the highest performance among those WOLEDs based on crystalline materials. CHM-TADFNA-D strategy demonstrates the great potential of organic crystalline materials for developing the next-generation WOLEDs.

## Materials and methods

Organic material BP1T was synthesized according to previous reports. 2FPPICz was commercially available from Jilin Yuanhe Electronic Material Company and purified twice by thermal gradient sublimation before using. DMAC-DPS was purchased from Xi’an Polymer Light Technology Corporation. BmPyPb and Ir(tptpy)_2_acac were bought from Luminescence Technology Corporation (Lumetc). These three materials were used as received.

### Film and device fabrication

The crystal thin film and devices were fabricated by WEG method, which can be realized by physical vacuum deposition as mentioned before. Heavily doped n-type Si/SiO_2_ substrates (capacitance per unit area, *C*_*i*_ = 10 nF cm^−2^) were applied to identify both the morphologies and crystal structures of crystalline films and nanoaggregates. Quartz substrates were used to investigate photoluminescence. Indium tin oxide (ITO) substrates, which have thickness of 180 nm and sheet resistance of 10 Ω were applied in fabrication of OLEDs and transient EL decay curves. ITO substrates were first purged with detergent. Next, the Si/SiO_2_, quartz, and ITO substrates were ultrasonically treated for 20 min with acetone, alcohol, and deionized water, respectively. Then they were together desiccated in high purity nitrogen and dried in a bake oven at 120 °C for 30 min. ITO was followed treated with oxygen plasma as well as spin-coated with PEDOT: PSS (Clevious P VP Al 4083) at 4000 rpm for 30 s and baked for 30 min at 120 °C. Afterward, the Si/SiO_2_, quartz, and ITO substrates were respectively transferred to the vacuum chamber at a pressure of under 10^−4^ Pa. BP1T and 2FPPICz were grown at speed of approximate 4 to 10 Å/min on a substrate of temperature of 102 °C. Afterwards, the substrate was cooled down to 40 °C, and the nanoaggregates were then grow at rate of 10 Å/min. Subsequently, the substrate was reheated to 102 °C and the CHM and dopants were grown at rates of 4 to 10 Å/min and 0.2 to 0.5 Å/min, respectively. Other CHM-NA-D layers also experienced the same process. The deposition rate of BmPyPb, LiF, LiCO_3_, and Al are 1–2 Å/s, 0.03–0.06 Å/s, 0.1–0.2 Å/s and 10–15 Å/s, respectively at room temperature. To identify the film thickness, a quartz-crystal microbalance was used. The effective emission area overlapping the area between the ITO and Al electrodes is 4.0 mm by 4.0 mm for each device.

### Film and device characterization

The morphologies of films and nanoaggregates were determined by a SPI 3800/SPA 300 HV (Seiko Instruments Inc., Japan) atomic force microscope with tapping mode. The out-of-plane XRD patterns were acquired using a Rigaku SmartLab X-ray diffraction instrument with Cu Ka radiation (*l* = 1.54056 Å). An Edinburgh Instrument FLS980 spectrometer was applied to measure the PL spectral, transient PL decay curves and transient EL decay curves. A Keithley source measurement system (Keithley 2400/2000) with a calibrated silicon photodiode and SpectraScan PR650 spectrophotometer were used to investigate the *J-V-L* characteristics of devices and electroluminescence spectra under an ambient atmosphere at room temperature. The EQE values were attained from current density, luminance and electroluminescence spectral by assuming a Lambertian distribution.

### Supplementary information

Supplementary Information

## Data Availability

The article includes all data analyzed during this study.
