# Supplementary material for: High-efficiency crystalline white organic light-emitting diodes
Source: Light Sci Appl. 2024 Apr 8;13:86. doi: 10.1038/s41377-024-01428-y (PMC11001915; doi:10.1038/s41377-024-01428-y)
Supplement: Supplementary file 1 — Supplementary Information [file 41377_2024_1428_MOESM1_ESM.docx]

**Supplementary Information For**

**High-Efficiency Crystalline White Organic Light-Emitting Diodes**

Yijun Liu^1, 2^, Feng Zhu^1, 2,*^, Yue Wang^3^, and Donghang Yan^1, 2^

*^1^State Key Laboratory of Polymer Physics and Chemistry Changchun Institute of Applied Chemistry Chinese Academy of Sciences, Changchun 130022, China;
^2^School of Applied Chemistry and Engineering, University of Science and Technology of China, Hefei 230026, China;*

*^3^State Key Laboratory of Supramolecular Structure and Materials, Jilin University, Changchun 130012, China;*

**Corresponding author:*

*Feng Zhu:* [*zhufeng@ciac.ac.cn*](mailto:zhufeng@ciac.ac.cn)*; (+86 431)85262234*


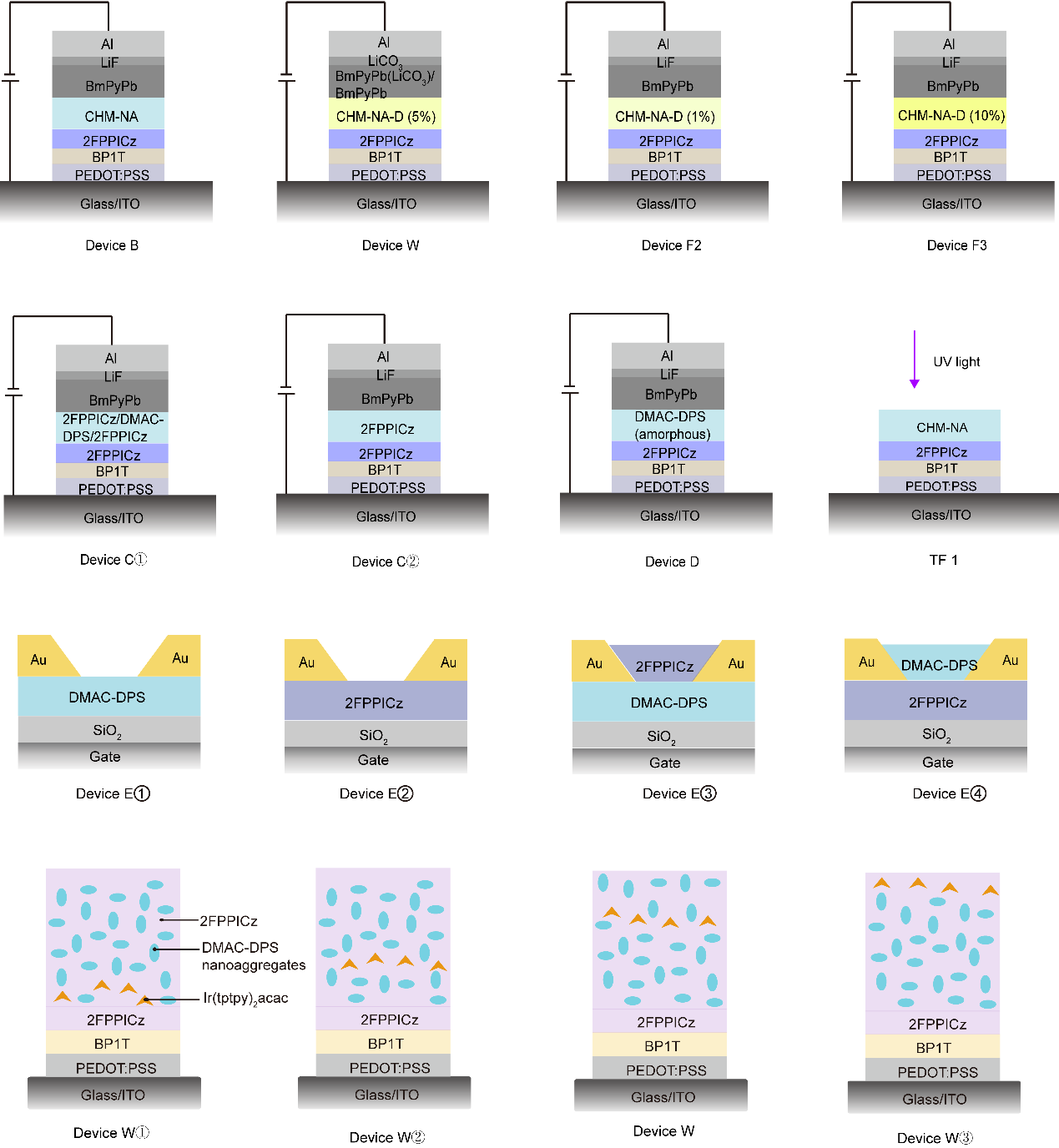


**Figure S1.** Device Structure Schematic.


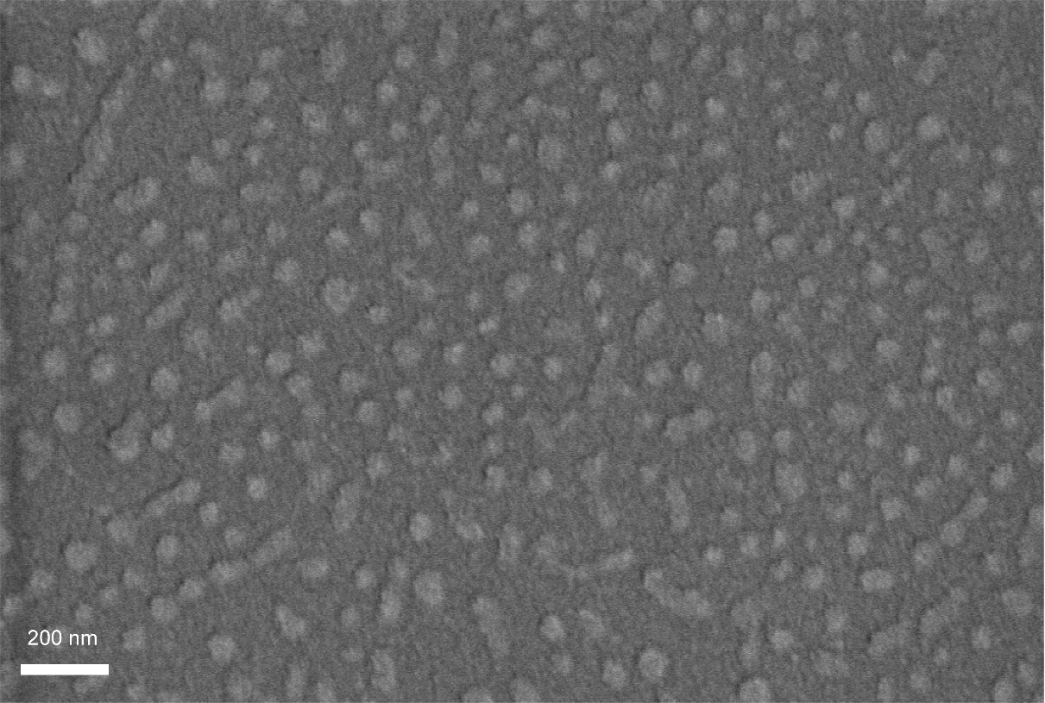


**Figure S2.** Scanning electron microscope (SEM) image of DMAC-DPS nanoaggregates.


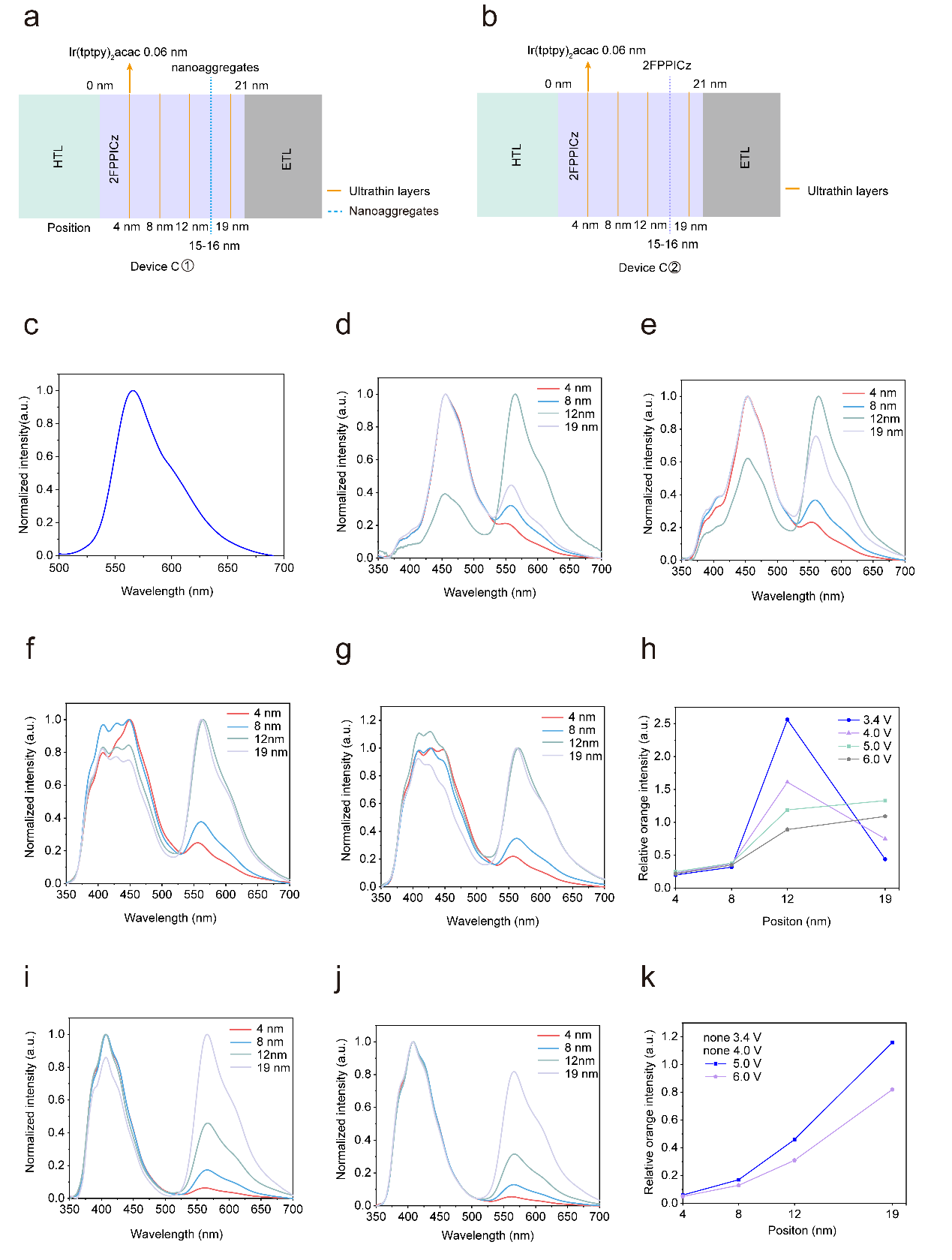


**Figure S3.** (a) Schematic illustration for confirming the different inserting positions (4, 8, 12, 19 nm) in Device C①. (b) Schematic illustration for confirming the different inserting positions (4, 8, 12, 19 nm) in Device C②. (c) PL spectrum of 2FPPICz: 5 wt% Ir(tptpy)_2_acac. (d) Normalized EL spectra of Device C① at 3.4 V. (e) Normalized EL spectra of Device C① at 4.0 V. (f) Normalized EL spectra of Device C① at 5.0 V. (g) Normalized EL spectra of Device C① at 6.0 V. (h) Exciton distribution of Device C①. (i) Normalized EL spectra of Device C② at 5.0 V. (j) Normalized EL spectra of Device C② at 6.0 V. (k) Exciton distribution of Device C①.

Device C① is composed of ITO/PEDOT:PSS (40 nm)/BP1T (7 nm)/2FPPICz (7 nm)/2FPPICZ (15 nm)/DMAC-DPS (1 nm)/2FPPICz (5 nm)/BmPyPb (40 nm)/LiF/Al. The Device C② has a similar structure, except for the 1 nm 2FPPICz being replaced by 1 nm DMAC-DPS. Briefly, Device C① represents a device with one layer of DMAC-DPS nanoaggregates, whereas Device C② is a pure 2FPPICz device. Ultrathin long wavelength phosphorescent material Ir(tptpy)2acac layers with the thickness of 0.06 nm were inserted into EMLs of both Device C① and Device C② at position of 4 nm, 8 nm, 12nm and 19 nm, respectively (Figure S2a-b). In accordance with the hole-transport characteristic of 2FPPICz, the exciton recombination region of Device C② is located close to the electron transport layer. By contrast, Device C①’s exciton recombination region is shifted to left, owing to the addition of bipolar DMAC-DPS nanoaggregates.


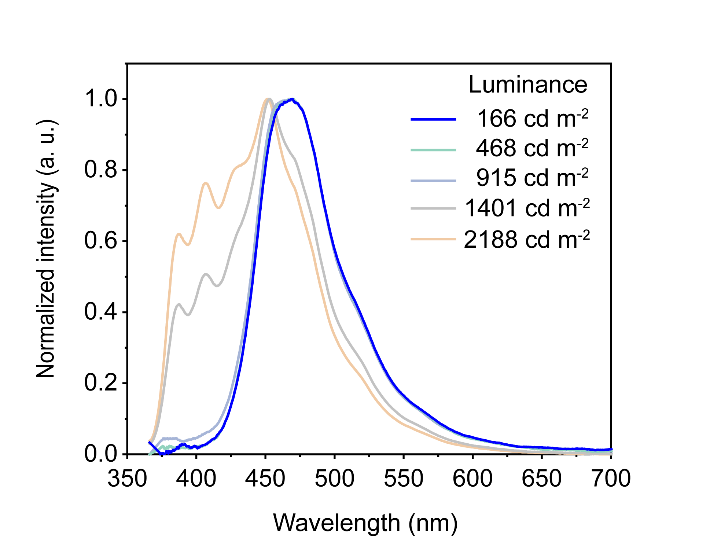


**Figure S4.** EL spectrum of Device B at varied voltages.


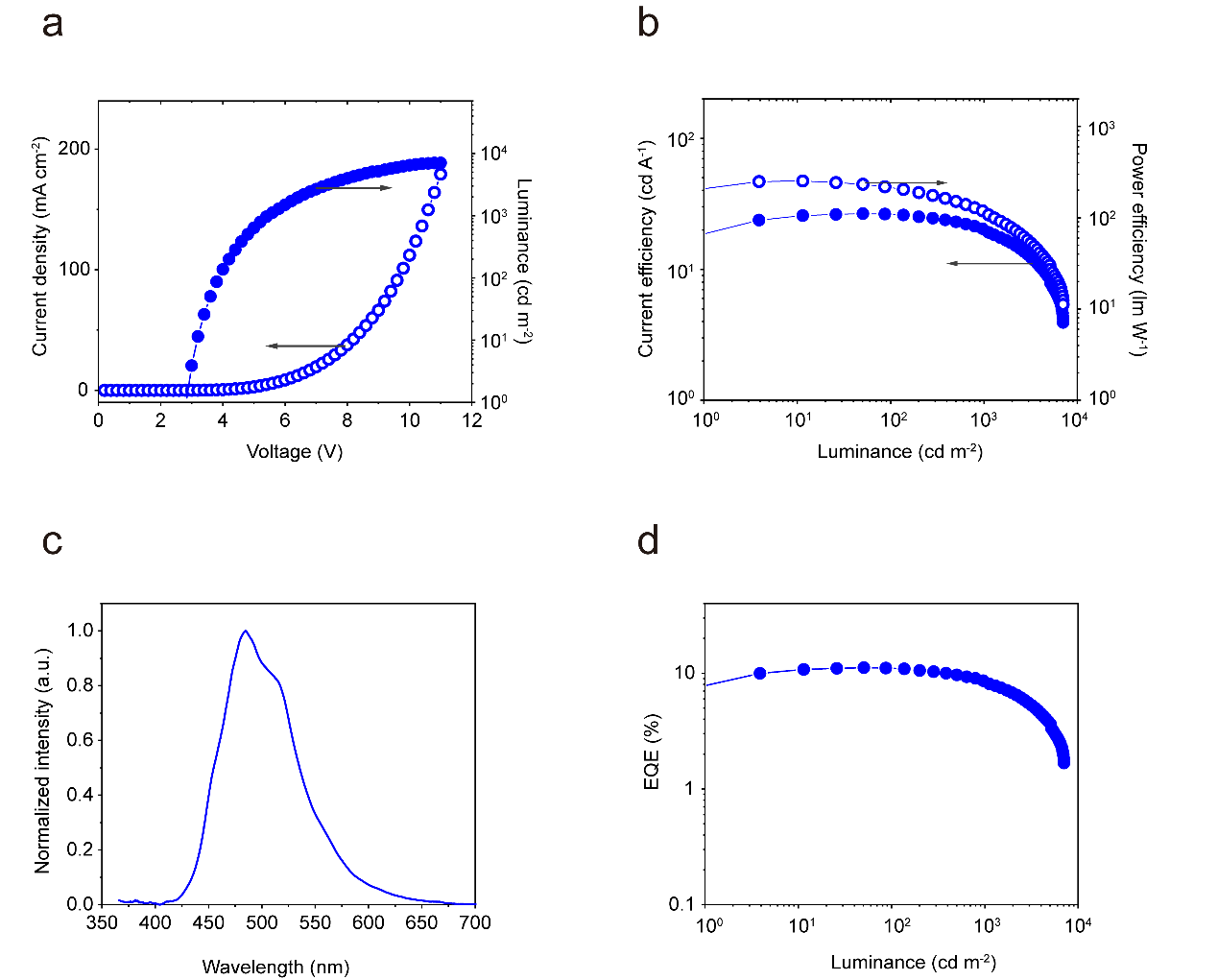


**Figure S5.** (A) Voltage dependent current density and luminance. (B) Luminance dependent CE and PE characteristics. (C) EL spectrum at varied voltages. (D) EQE-luminance curve of Device D.


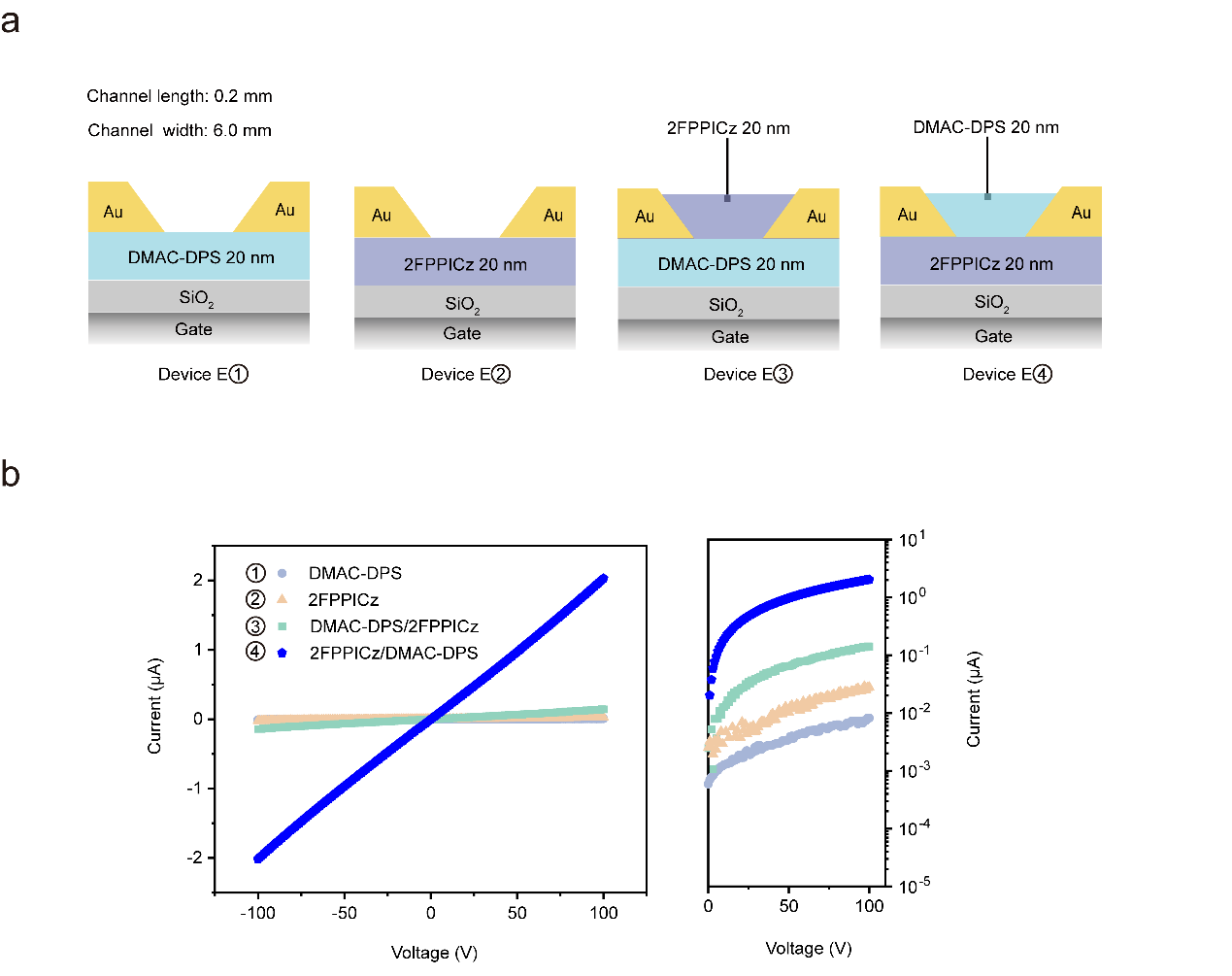


**Figure S6.** (a) Structures of in-plane transport devices. (b) Voltage-dependent current of in-plane transport devices.

Device E①: amorphous DMAC-DPS thin film (20 nm);

Device E②: amorphous 2FPPICz thin film (20 nm);

Device E③: amorphous DMAC-DPS thin film (20 nm)/amorphous 2FPPICz thin film (20 nm);

Device E④: amorphous 2FPPICz thin film (20 nm)/amorphous DMAC-DPS thin film (20 nm).

The channel length and channel width are 0.2 mm and 6.0 mm, respectively. At the two terminals of the Au electrode, a bias voltage (-100 V ~100 V) is applied to measure the produced currents of each device.


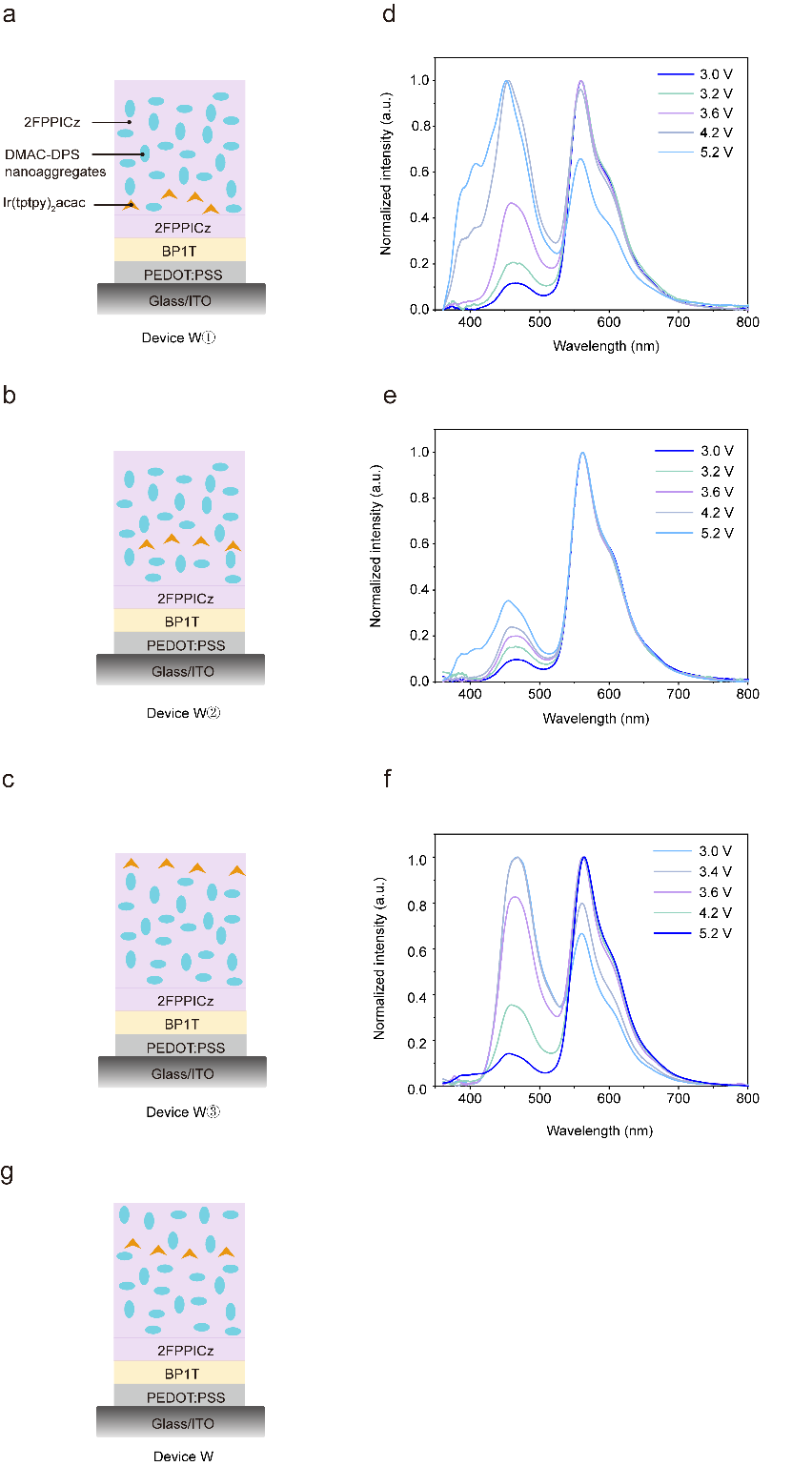


**Figure S7.** Schematic illustration of doping position in (a) First five-nanometer region (Device W①), (b) Second five-nanometer region (Device W②), (c) Fourth five-nanometer region (Device W③). EL spectrum at varied voltages for Ir(tptpy)_2_acac doped in different CHM layers. (d) for Device W①, (e) for Device W②, (f) for Device W③. (g) Schematic illustration of doping position in the third five-nanometer region (Device W) for reference.

Three additional devices, Device W①, Device W② and Device W③, with phosphorescent material doping in different positions of first, second and forth five-nanometer regions in CHM were fabricated respectively (Figure S6a-c). As Figure S6df shown, except for the third five-nanometer region (Device W), all of devices doping in other three layers exhibit changeable spectrum property.


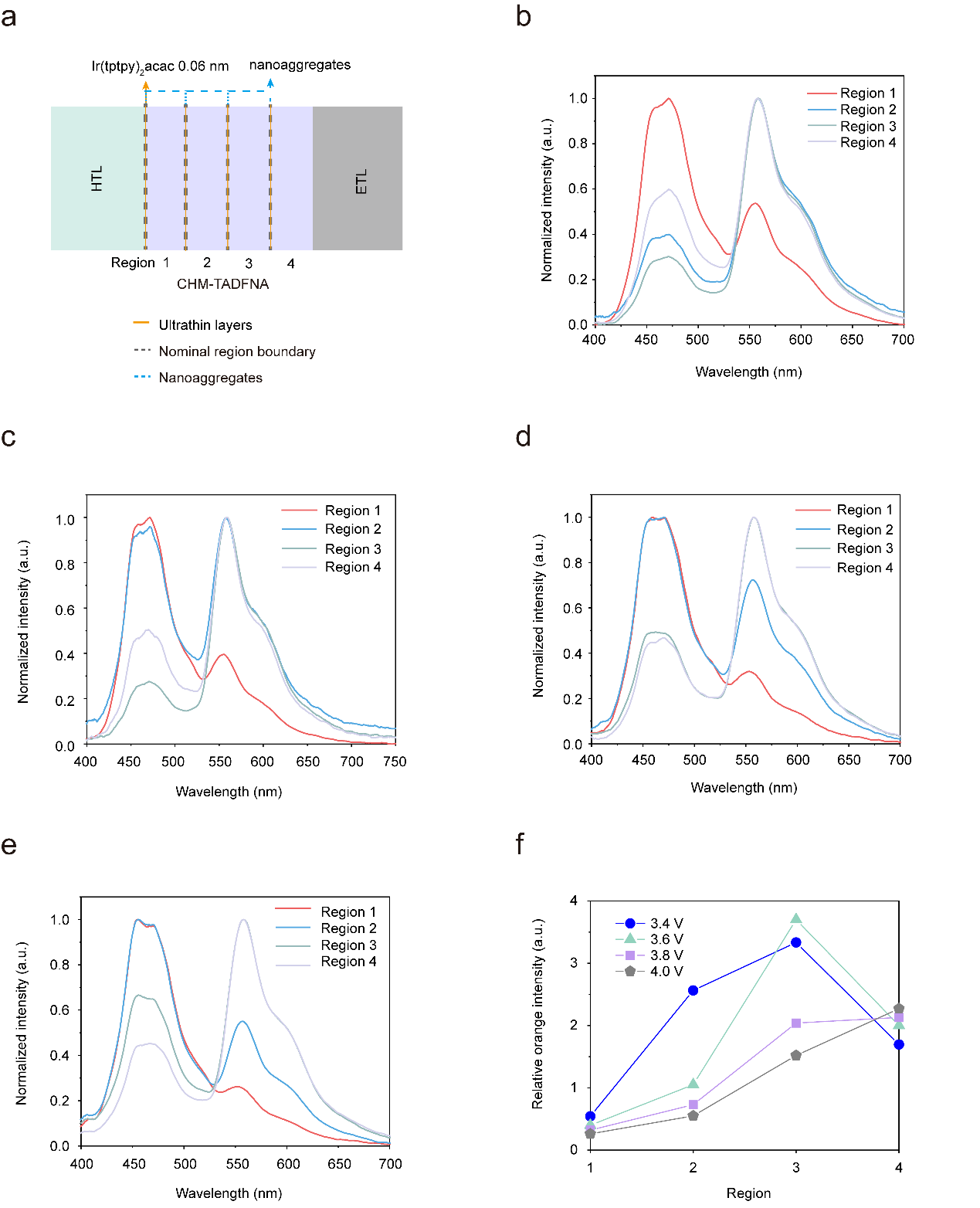


**Figure S8.** Exciton distribution in Device B. (a) Schematic illustration for confirming the different inserting positions (Region 1, 2, 3, 4) in Device B. (b) Normalized EL spectra of Device B at 3.4 V. (c) Normalized EL spectra of Device B at 3.6 V. (d) Normalized EL spectra of Device B at 4.0 V. (e) Normalized EL spectra of Device B at 5.0 V. f) Normalized EL spectra of Device B at 6.0 V. (g) Exciton distribution of Device B at different voltages.
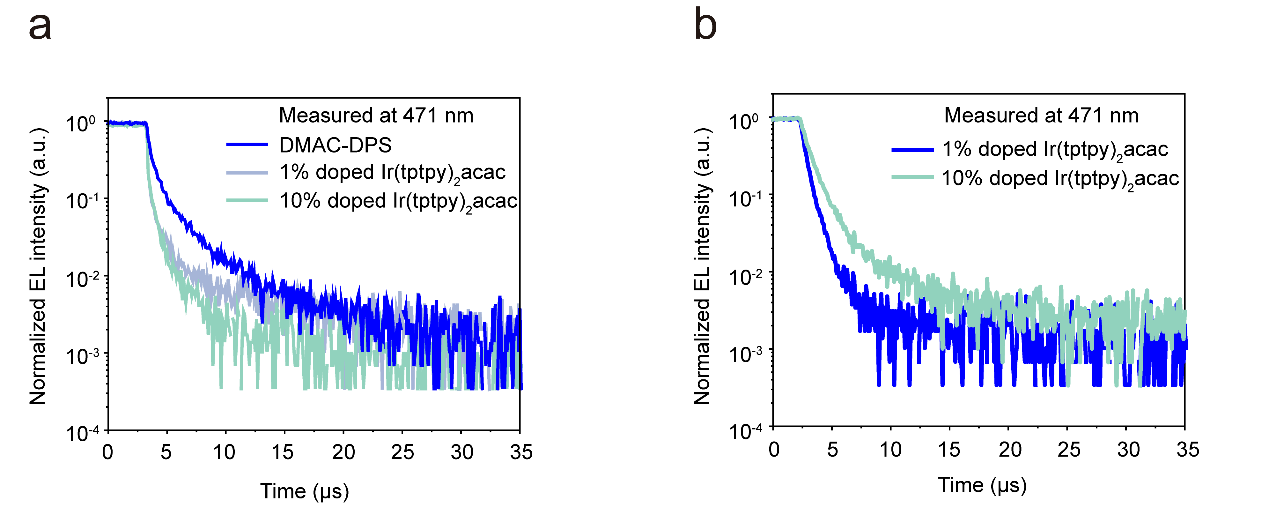


**Figure S9.** (a) EL decay transient curves of 471 nm emission bands of Device F1, Device F2 and Device F3. (b) EL decay transient curves of 555 nm emission bands of Device F2 and Device F3.


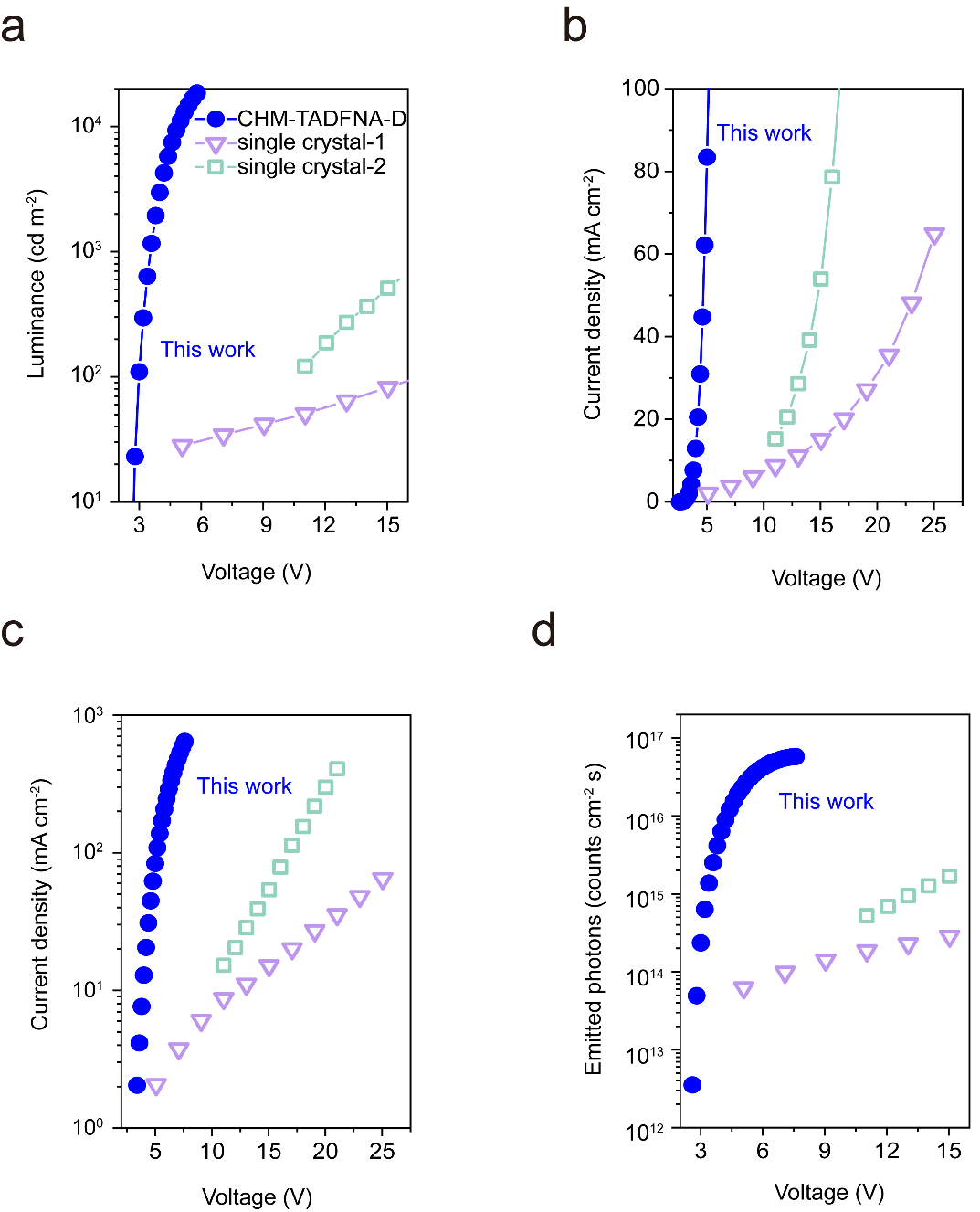


**Figure S10.** Comparisons of CHM-TADFNA-D WOLED with Single crystal WOLEDs. (A) A comparison of voltage (*V*)-luminance and (B) voltage (*V*)-dependent current density (*J*). (C) Comparison of voltage-dependent semi-log density and (D) Comparison of voltage(*V*)-dependent semi-log emitted photons (*N*), between the CHM-TADFNA-D WOLED and reported single crystal WOLEDs^1,2^. All reference data for comparison are extracted from the corresponding literature.

**Supplementary references**

1 Zhu, Q.-C. et al. Enhanced performance of white organic light-emitting devices based on ambipolar white organic single crystals. *Applied Physics Letters* 118, 163301 (2021).

2 Ding, R. et al. High-Color-Rendering and High-Efficiency White Organic Light-Emitting Devices Based on Double-Doped Organic Single Crystals. *Advanced Functional Materials* 29, 1807606 (2019).
